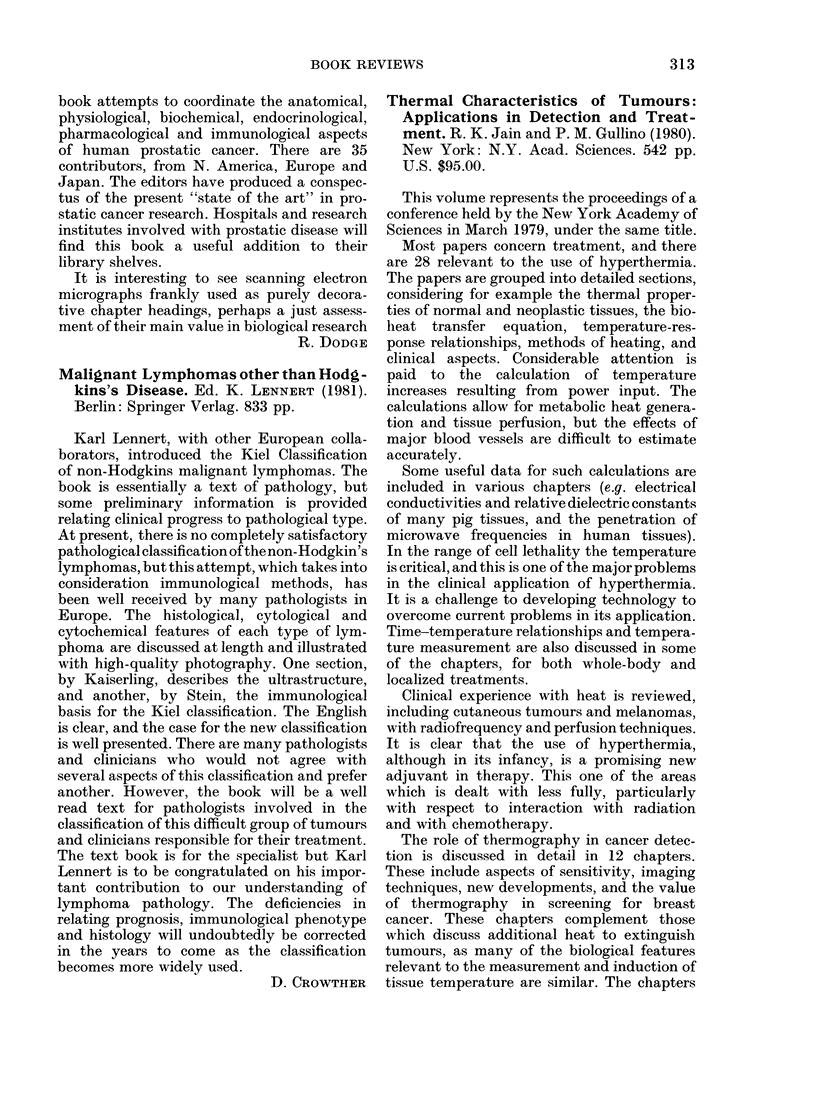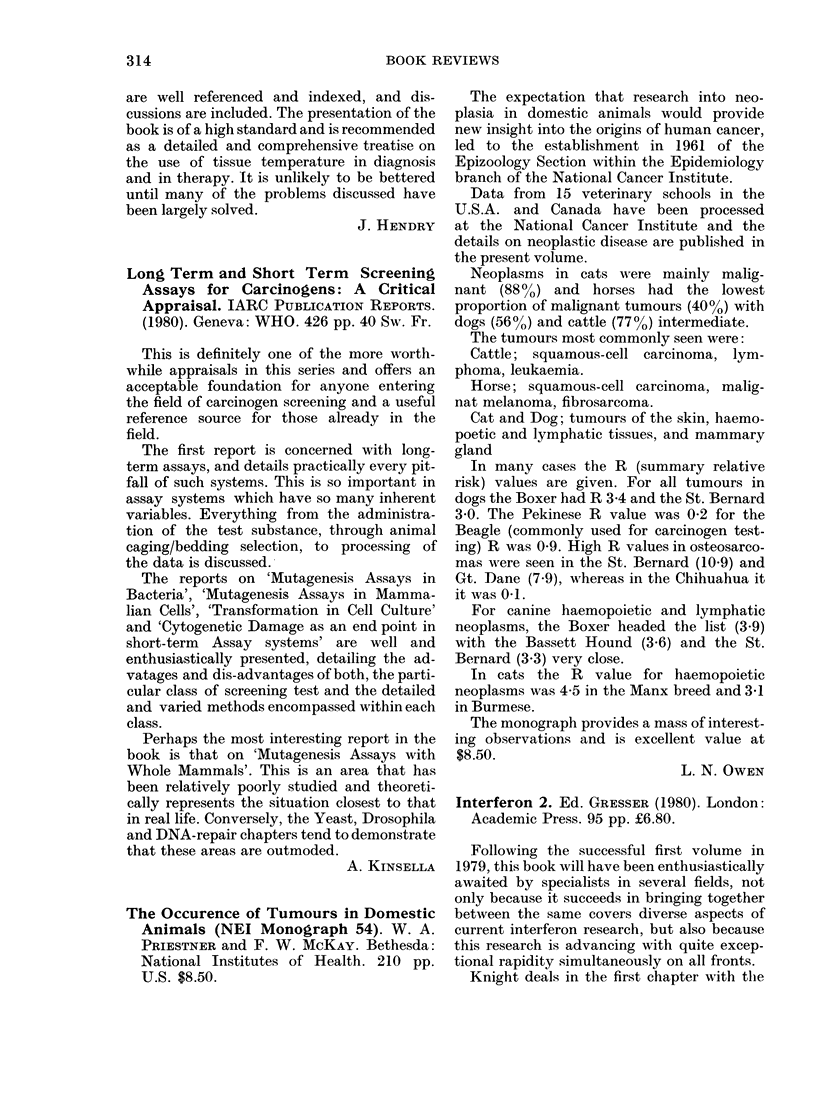# Thermal Characteristics of Tumours: Applications in Detection and Treatment

**Published:** 1981-08

**Authors:** J. Hendry


**Thermal Characteristics of Tumours: Applications in Detection and Treatment.** R. K. Jain and P. M. Gullino (1980). New York: N.Y. Acad. Sciences. 542 pp. U.S. $95.00.

This volume represents the proceedings of a conference held by the New York Academy of Sciences in March 1979, under the same title.

Most papers concern treatment, and there are 28 relevant to the use of hyperthermia. The papers are grouped into detailed sections, considering for example the thermal properties of normal and neoplastic tissues, the bioheat transfer equation, temperature-response relationships, methods of heating, and clinical aspects. Considerable attention is paid to the calculation of temperature increases resulting from power input. The calculations allow for metabolic heat generation and tissue perfusion, but the effects of major blood vessels are difficult to estimate accurately.

Some useful data for such calculations are included in various chapters (*e.g.* electrical conductivities and relative dielectric constants of many pig tissues, and the penetration of microwave frequencies in human tissues). In the range of cell lethality the temperature is critical, and this is one of the major problems in the clinical application of hyperthermia. It is a challenge to developing technology to overcome current problems in its application. Time–temperature relationships and temperature measurement are also discussed in some of the chapters, for both whole-body and localized treatments.

Clinical experience with heat is reviewed, including cutaneous tumours and melanomas, with radiofrequency and perfusion techniques. It is clear that the use of hyperthermia, although in its infancy, is a promising new adjuvant in therapy. This one of the areas which is dealt with less fully, particularly with respect to interaction with radiation and with chemotherapy.

The role of thermography in cancer detection is discussed in detail in 12 chapters. These include aspects of sensitivity, imaging techniques, new developments, and the value of thermography in screening for breast cancer. These chapters complement those which discuss additional heat to extinguish tumours, as many of the biological features relevant to the measurement and induction of tissue temperature are similar. The chapters are well referenced and indexed, and discussions are included. The presentation of the book is of a high standard and is recommended as a detailed and comprehensive treatise on the use of tissue temperature in diagnosis and in therapy. It is unlikely to be bettered until many of the problems discussed have been largely solved.

J. HENDRY